# Astrocytes and pericytes attenuate severely injured patient plasma mediated expression of tight junction proteins in endothelial cells

**DOI:** 10.1371/journal.pone.0270817

**Published:** 2022-07-05

**Authors:** Preston Stafford, Sanchayita Mitra, Margot Debot, Patrick Lutz, Arthur Stem, Jamie Hadley, Patrick Hom, Terry R. Schaid, Mitchell J. Cohen

**Affiliations:** Division of GITES, Department of Surgery, University of Colorado Anschutz Medical Campus, Aurora, Colorado; Eötvös Loránd Research Network Biological Research Centre, HUNGARY

## Abstract

Blood Brain Barrier (BBB) breakdown is a secondary form of brain injury which has yet to be fully elucidated mechanistically. Existing research suggests that breakdown of tight junction proteins between endothelial cells is a primary driver of increased BBB permeability following injury, and intercellular signaling between primary cells of the neurovascular unit: endothelial cells, astrocytes, and pericytes; contribute to tight junction restoration. To expound upon this body of research, we analyzed the effects of severely injured patient plasma on each of the cell types in monoculture and together in a triculture model for the transcriptional and translational expression of the tight junction proteins Claudins 3 and 5, (CLDN3, CLDN5) and Zona Occludens 1 (ZO-1). Conditioned media transfer studies were performed to illuminate the cell type responsible for differential tight junction expression. Our data show that incubation with 5% human *ex vivo* severely injured patient plasma is sufficient to produce a differential response in endothelial cell tight junction mRNA and protein expression. Endothelial cells in monoculture produced a significant increase of CLDN3 and CLDN5 mRNA expression, (3.98 and 3.51 fold increase vs. control respectively, p<0.01) and CLDN5 protein expression, (2.58 fold change vs. control, p<0.01), whereas in triculture, this increase was attenuated. Our triculture model and conditioned media experiments suggest that conditioned media from astrocytes and pericytes and a triculture of astrocytes, pericytes and endothelial cells are sufficient in attenuating the transcriptional increases of tight junction proteins CLDN3 and CLDN5 observed in endothelial monocultures following incubation with severely injured trauma plasma. This data suggests that inhibitory molecular signals from astrocytes and pericytes contributes to prolonged BBB breakdown following injury via tight junction transcriptional and translational downregulation of CLDN5.

## Introduction

Traumatic Brain Injury (TBI) is one of the leading causes of mortality worldwide, with mortality rates as high as 30% and significant morbidity in survivors [1,2]. Following trauma, treating primary brain injury and mitigating secondary brain injury is the primary focus. Separate from direct TBI, non-brain injured trauma patients also display brain injury like symptomatology. In both cases, (direct TBI and indirect TBI) microvascular injury and neuroinflammation are thought to drive this pathology and have become a target for research and therapeutics [3–5]. A key component of this microvascular inflammatory injury is breakdown of the Blood Brain Barrier (BBB) which is associated with morbidity and mortality following TBI [4], and observationally following severe non-TBI traumatic injury. Central to targeted therapeutic treatment for BBB dysfunction is understanding the pathology underlying cellular responses to traumatic injury. In this we look to address brain pathology in non-TBI injured patients, by examining BBB function after non-TBI trauma.

The Blood Brain Barrier regulates transcytotic movement of molecules between the vasculature and neuronal space. The BBB is composed of the neurovascular unit, comprising three main cell types–endothelial cells, astrocytes, and pericytes. These cell types participate in the formation of a physical barrier composed of tight junction proteins Claudin 3 (CLDN3), Claudin 5 (CLDN5), Occludin, Junctional Adhesion Molecules (JAM1, JAM2, JAM3), and the anchor proteins Zona Occludens 1, 2, and 3, (ZO-1, ZO-2, ZO-3) expressed and localized within and between the endothelial cells [5]. This physical barrier is the principle homeostatic regulator between the CNS and peripheral vasculature [6] and performs the essential functions of facilitating the transfer of nutrients, regulating ion stasis, and blocking noxious molecules from flowing into the neuronal extracellular space [5]. BBB dysfunction results in adverse patient outcomes linked to transcytotic leakage of fluids, proteins, and other molecules, leading to intraneuronal toxicity and homeostatic imbalance [7–9].

Central to BBB integrity is the maintenance of tight junction proteins which can be degraded through protease activity following TBI [10]. BBB degradation and subsequent leak of inflammatory soluble factors into intraneuronal space has been demonstrated to occur as soon as 30 minutes post-injury, with maximal leakage occurring in a biphasic manner starting as early as 4 hours post-injury, and continued permeability up to 30 days post-injury [11–13]. Based on these findings [6,14–18] we focused on the expression of three tight junction proteins essential to BBB permeability maintenance, CLDN3, CLDN5 and ZO-1.

To elucidate this mechanism in the context of trauma, we incubated cells in monoculture and in triculture with severely injured patient plasma and performed conditioned media exchange studies to probe the contribution of each cell type to tight junction expression. We hypothesized that plasma from non-TBI traumatically injured patients leads to a loss of junctional integrity between endothelial cells via transcriptional down regulation of the major gap junction proteins Claudins 3, 5 and ZO-1.

## Methods

### Patient plasma

Normal pooled plasma (George King Bio-medical) phenotypes were verified by the manufacturer and used as our healthy plasma negative control. Experimental plasma was collected from severely injured trauma patients upon arrival to the emergency department at a Level 1 trauma center. Plasma samples were collected, and patient demographics described in accordance with a sampling protocol approved by the Colorado Multiple Institutional Review Board (COMIRB#13–3087); all subjects were informed, and collection performed under waiver of consent. (Tables 1 and 2) [19] Patient demographics were collected, and injury severity score and base deficit was assessed [20–22]. To address limited ex vivo plasma volumes, two separate groups of pooled trauma plasma were created by combining individual patient samples. The severely injured patient plasma was pooled based upon the patient’s injury severity score (ISS), and base deficit (BD). ISS is used to determine the extent of injury severity, and BD is used to determine tissue hypoperfusion [20–22]. Severe injury is any sample from a patient with an ISS>15 and BD<-6.

**Table 1:** Trauma plasma pool for transcription experiments described in Figs 1 and 2.

Trauma Plasma Pool	Age	ISS	BD	Blunt/Penetrating
1	62	38	-9.7	Blunt
1	40	25	-12.7	Blunt
1	43	25	-24.7	Blunt
1	35	34	-11	Blunt
1	62	24	-8.3	Blunt
1	23	41	-7	Blunt
1	24	66	-10	Penetrating
1	38	25	-9.3	Blunt
1	38	43	-7	Penetrating
1	23	42	-6.3	Penetrating
1	36	17	-10	Penetrating
1	41	19	-7	Penetrating
1	50	16	-10	Blunt
1	38	29	-13	Blunt
1	26	25	-10	Penetrating
1	37	26	-13.6	Penetrating
1	34	42	-25	Blunt
1	24	33	-12.4	Penetrating
1	19	25	-10.3	Blunt
1	55	29	-13	Blunt
**Median**	37.5	27.5	-10.0	

**Table 1-** Pooled plasma samples from severely injured patients used for transcription experiments in Figs 1 and 2. Samples were comprised of equivalent volumes from each patient pooled together, 500 μL from each patient. Both individual patients and final pooled plasma were selected with the cutoff criteria of ISS<15, BD<-6.

**Table 2:** Trauma plasma pool used for ELISA.

Trauma Plasma Pool	Age	ISS	BD	Blunt/Penetrating
2	33	34	-6	Blunt
2	44	22	-13	Blunt
2	28	29	-17	Blunt
2	24	59	-13	Blunt
2	31	16	-11	Blunt
2	27	30	-19.3	Penetrating
2	57	75	-27.5	Penetrating
2	54	25	-24.5	Blunt
2	43	25	-24.7	Penetrating
2	36	41	-27.6	Penetrating
**Median**	34.5	29.50	-18.2	

**Table 2-** Pooled plasma samples from severely injured patients used for translation experiments in Figs 3 and 4. Samples were comprised of equivalent volumes from each patient pooled together, 500 μL from each patient. Both individual patients and final pooled plasma were selected with the cutoff criteria of ISS<15, BD<-6.

### Cell culture

Immortalized Human Cerebral Microvascular Endothelial cells (hCMEC/D3) and complete cell growth media (EndoGRO-MV) were obtained from EMD Millipore and cultured as described by the manufacturer. Primary human astrocytes with complete astrocyte media and primary human brain vascular pericytes with complete pericyte growth media were purchased from Science Cell and cultured in astrocyte and pericyte specific media respectively, as described by the manufacturer. Purity and phenotype of each cell type were verified by manufacturer with certificate of analysis provided upon delivery. HCMEC/D3 cells cultured with less than 12 passages from passage indicated by manufacturer were used for all experiments. Human astrocytes and pericytes cultured with less than 8 passages from passage indicated by manufacturer were used for all experiments.

Triculture Model on Insert: The triculture model was an adaptation of previously published protocols from Hatherell et. al. 2011 and Stone et. al. 2019 [23,24]. In brief, transwell 12 well cell inserts with a 0.4 μm 0.9 cm^2^ PET membrane (Falcon) were coated for one hour with a 50–50 mixture of 1% rat-tail Collagen Type II and 1% Poly-L-Lysine on the basal surface. The coating was left applied for an hour before being removed by vacuum pipette. To distinguish between the apical and basal (top and bottom) surface of the cell insert membrane, we denoted them as follows. The membrane facing up inside the well of the insert is denoted as apical. The membrane facing down on the outside of the insert exposed to the culture well plate is denoted as basal.

For monocultures, endothelial cells were plated on the apical membrane at a concentration of 300k cells; The Pericytes and Astrocytes were plated on the basal membrane at a concentration of 300k and 100k respectively. Astrocytes and pericytes plated on the basal side were supplemented with additional media to a volume total 300μL. For the triculture, the basal side was plated first, with pericytes added first and given 30min to adhere, followed by the addition of astrocytes and another 30 min incubation to allow for adherence of the cells to the membrane. Following the one-hour incubation, the cell inserts were inverted and placed into a 12 well cell culture plate. For the triculture, following the inversion into the 12 well culture plate, 300K endothelial cells were plated on the apical surface. The apical surface was supplemented with 1 mL growth media and the basal surface with 3 mL of growth media and incubated for 48 hours in a humidified chamber at 37 C⁰ with 5% CO_2_.

After the incubation period of 48 hours, media was removed from the culture wells and fresh media supplemented with 5% severely patient plasma or healthy plasma was added to the apical side of the insert and incubated for 4 or 6 hours. Cells sub-cultured for each experiment were supplemented with media according to manufacturer protocol. Following seeding of cells on inserts for each experiment and in order to avoid alterations in results due to differences in media type, all experimental cell cultures were supplemented with a 1:1:1 mixture of endothelial, astrocyte, and pericyte media. The severely injured patient plasma for each experimental replicate was composed of pooled patient plasma from patients with non-TBI traumatic injuries ISS> 15 and BD<-6.

Conditioned Media (CM) Model: Endothelial, astrocyte, and pericyte monocultures, and tricultures were incubated with or without 5% severely injured patient plasma for 1 hour to produce cell type specific conditioned media. Conditioned media in the presence and absence of trauma plasma respectively (CM+TP; CM-TP) from each cell type was harvested. In order to interrogate the effect of other cell types on endothelial cells that have been exposed to severely injured patient plasma; Endothelial monocultures were supplemented with severely injured patient plasma to 5% working concentration followed immediately by supplementation of cell type specific conditioned media (CM+TP; CM-TP) to 10% CM working concentration. Naïve control endothelial cells were allowed to grow in their normal growth media throughout the experiment in absence of conditioned media and plasma.

### RNA extraction and cDNA synthesis

RNA extraction was performed using a Qiagen RNeasy extraction kit. RNA samples were concentrated using a speed vac, 40 C⁰ for 60 minutes, and quantified spectrophotometrically for cDNA synthesis using a Biotek Synergy H1 microplate reader. 1.5 μg total RNA was used for each cDNA synthesis reaction with QuantaBios qScript cDNA supermix using an adapted form of QuantaBios protocol. 60uL reactions were performed using the following protocol run on a Bio-Rad thermal cycler: 5 minutes at 25 C⁰, 30 min at 42 C⁰, 5 min at 85 C⁰, cool down and hold at 4 C⁰.

### qPCR

qPCR protocols were adapted and optimized from recommended Quantstudios protocols. All RT-qPCR was performed on a QuantStudio 3 Real-Time PCR System (Applied Biosystems). All primer probe pairs were purchased from Applied Biosystems- GAPDH (Hs99999905_m1, Assay Location 229), CLDN3 (Hs00265816_s1, Assay Location 807), CLDN5 (Hs00533949_s1, Assay Location 1713), and ZO-1 (Hs01551861_m1, Assay Location 1762) for TaqMan based qPCR. The master mix used was comprised of 10uL of TaqMan fast advanced master mix (Applied Biosystems), 1uL of combined primer probes, and 3uL of RNAse free water per reaction. 6uL cDNA mix from cDNA synthesis was added to each well containing 14uL of master mix. The 20uL reactions were run on a 96 well plate in a Quantstudios 3 RT-PCR system with the following protocol: 2 minutes at 50 C, 10 minutes at 95 C, 20 seconds at 95 C and 1 minute at 60 C for 40 cycles. Ct values of the gene of interest were normalized to endogenous GAPDH control. Fold changes were produced from normalized Ct values compared to normalized experimental controls

### Whole cell lysate processing

Following the removal of the media, each cell insert was washed with 1X PBS. Cells were then trypsinized with 0.25% Trypsin EDTA for 10 mins. The cell pellet was collected by centrifugation at 100 g for 5 mins. Pellets were washed in 1X PBS three times and suspended in 50 μL 1X PBS. The whole cell lysate (WCL) was prepared as follows. The suspended pellets were placed in a Diagenode Biorupter for 7.5 minutes with sustained pulse intervals, 30 second on, 30 second off. The Lysate was then spun down at 10,000 rpm for 5 minutes. The supernatant was collected, and the pellet discarded. Protein concentrations in cell lysates were measured using Bradford assay. A standard curve was created by diluting 2mg/mL of BSA to 50, 25, 20, 10, 5, and 2.5 ug/mL in nanopure water. Samples were diluted 1:150; 150uL of the standards and diluted sample were added to a flat bottom 96 well plate in duplicate along with 150uL of Coomassie plus protein reagent and incubated at room temperature for 10 min. Optical Density was measured at 595 nm using a Biotek Synergy H1 microplate reader. Protein concentration was determined using a 4 parametric nonlinear regression standard curve.

### ELISA

Quantification of CLDN3 and CLDN5 in whole cell lysates were performed using a sandwich ELISA kit in accordance with the manufacturer’s protocol. (Antibodies Online Sandwich ELISA Assay Kits for CLDN3, ABIN6954828, and CLDN5, ABIN6962352) 4ug of WCL per sample was prepared and diluted in 200uL of PBS and added to the ELISA wells per manufacturer’s protocol. Optical Density was measured using a Biotek Synergy H1 microplate reader at 450 nm. A 4-parametric nonlinear regression standard curve was used to determine protein concentration. The fold change was normalized against control samples.

### Data analysis

GraphPad Prism 9 and Microsoft Excel 2016 were used for statistical analysis. qRT-PCR gene expression analysis was calculated using the 2^-ΔΔCt^ method (delta-delta Ct method). Control values were set to a value of 1 compared to sample values following 2^-ΔΔCt^ calculations. All data was represented as fold change over control. Statistical analysis was performed with GraphPad Prism 9 software using a two-way ANOVA test with Tukey’s multiple comparison post-hoc analysis. Experimental samples for non-conditioned media runs were compared against their own naïve controls. Experimental samples for conditioned media experiments were compared to both their naïve control and to each condition type respectively (CM+TP; CM-TP). *P* values of less than 0.05 were considered statistically significant.

## Results

### Severely injured patient plasma induces differential transcription expression of CLDN3 and CLDN5

**Fig 1:**
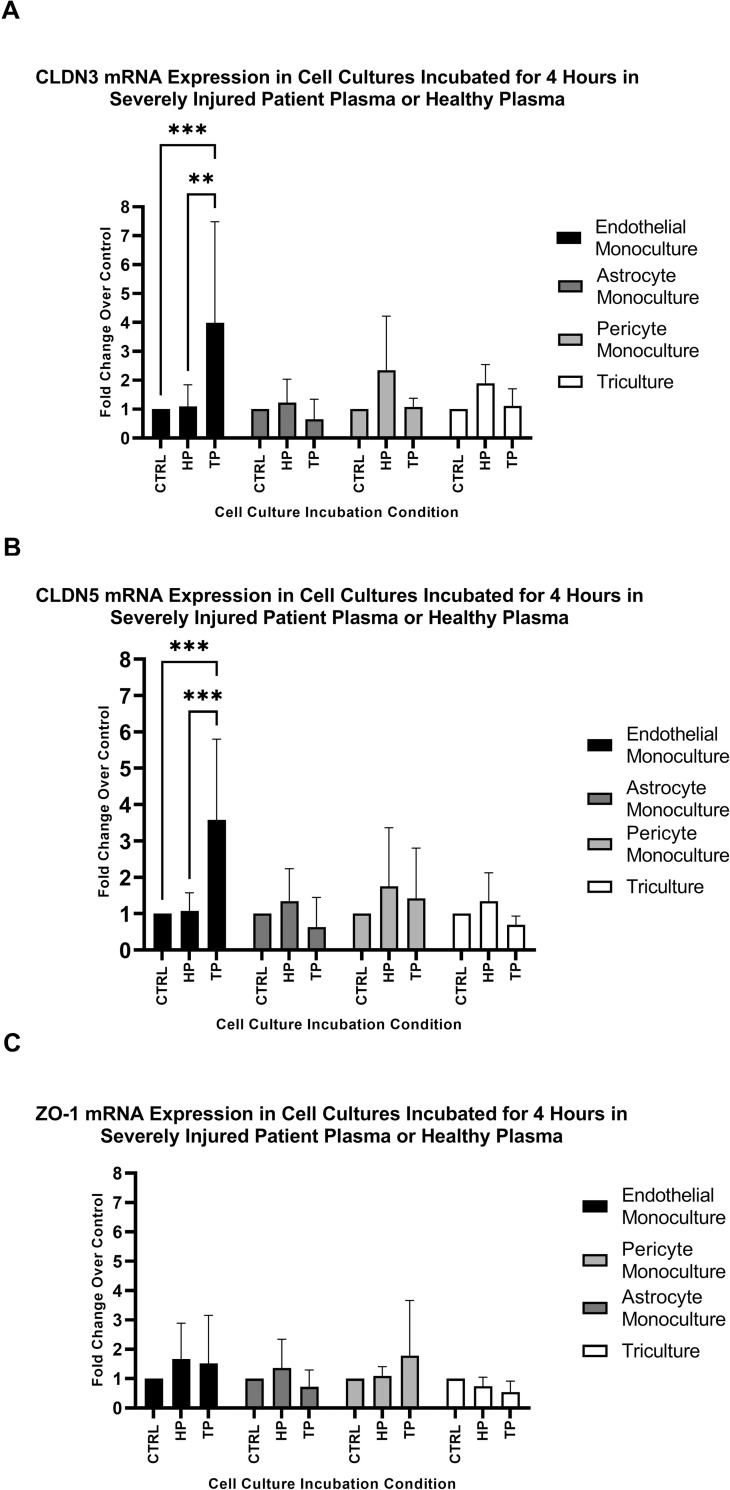
CLDN5, CLDN3, and ZO-1 mRNA expression following a 4 hour incubation with a working concentration of 5% plasma from healthy patients (HP) or 5% Trauma Plasma from severely injured patients (TP). (A) CLDN3 mRNA expression: The Endothelial monoculture incubated with TP showed a 3.98-fold increase vs. control, p<0.001, n = 6; Astrocyte monocultures, Pericyte monocultures, and the triculture did not show any significant change vs. control. All cell cultures incubated with HP showed no significant change vs. control. (B) CLDN5 mRNA expression: Endothelial monoculture showed a 3.51-fold increase vs. control, p<0.001, n = 6; Astrocyte monocultures, Pericyte monocultures, and the triculture did not show any significant fold change vs. control. All cell cultures incubated with HP showed no significant change vs. control. (C) ZO-1 mRNA expression: ZO-1 mRNA expression did not change significantly vs. control for either the monoculture or tricultures, n = 5. All cell cultures incubated with HP showed no significant change vs. control. Six experimental replicates were performed n = 6. ** Denotes p<0.01 *** Denotes p<0.001.

We initially examined transcriptional responses of junctional proteins in the BBB in response to *ex vivo* trauma plasma. The pooled plasma used in the transcription studies had a mean ISS of 31.2 and BD of -11.5. Taqman based quantitative RT-PCR was used to determine the transcriptional expression of CLDN3, CLDN5, and ZO-1. Endothelial, astrocyte, and pericyte monocultures, and the triculture showed no significant change in mRNA transcriptional expression of CLDN3, CLDN5, or ZO-1 following incubation with healthy plasma (Fig 1A–1C) Endothelial monocultures, had significant increases in transcriptional expression of tight junction protein CLDN3 (3.98 fold increase vs. control, p<0.001) and CLDN5 (3.6 fold increase vs. control, p<0.001) (Fig 1A and 1B) following a four hour incubation with trauma plasma from severely injured patients. This fold increase vs. control in expression of CLDN3 was not observed in astrocyte monoculture, pericyte monoculture, or the triculture. (Fig 1A) Similarly, CLDN5 transcriptional response also did not change vs. control values in astrocyte monoculture, pericyte monoculture, or the triculture. (Fig 1B) ZO-1 expression did not show any significant change vs. control for cells grown in monoculture or in the triculture model. (Fig 1C) To further expound upon these findings, a conditioned media experiment was performed to delineate the specific cell types contributing to the downregulation of the tight junction proteins in the triculture vs. the endothelial monoculture.

### Addition of astrocyte, pericyte, or triculture conditioned media suppress endothelial monoculture transcriptional upregulation of CLDN3 and CLDN5

**Fig 2:**
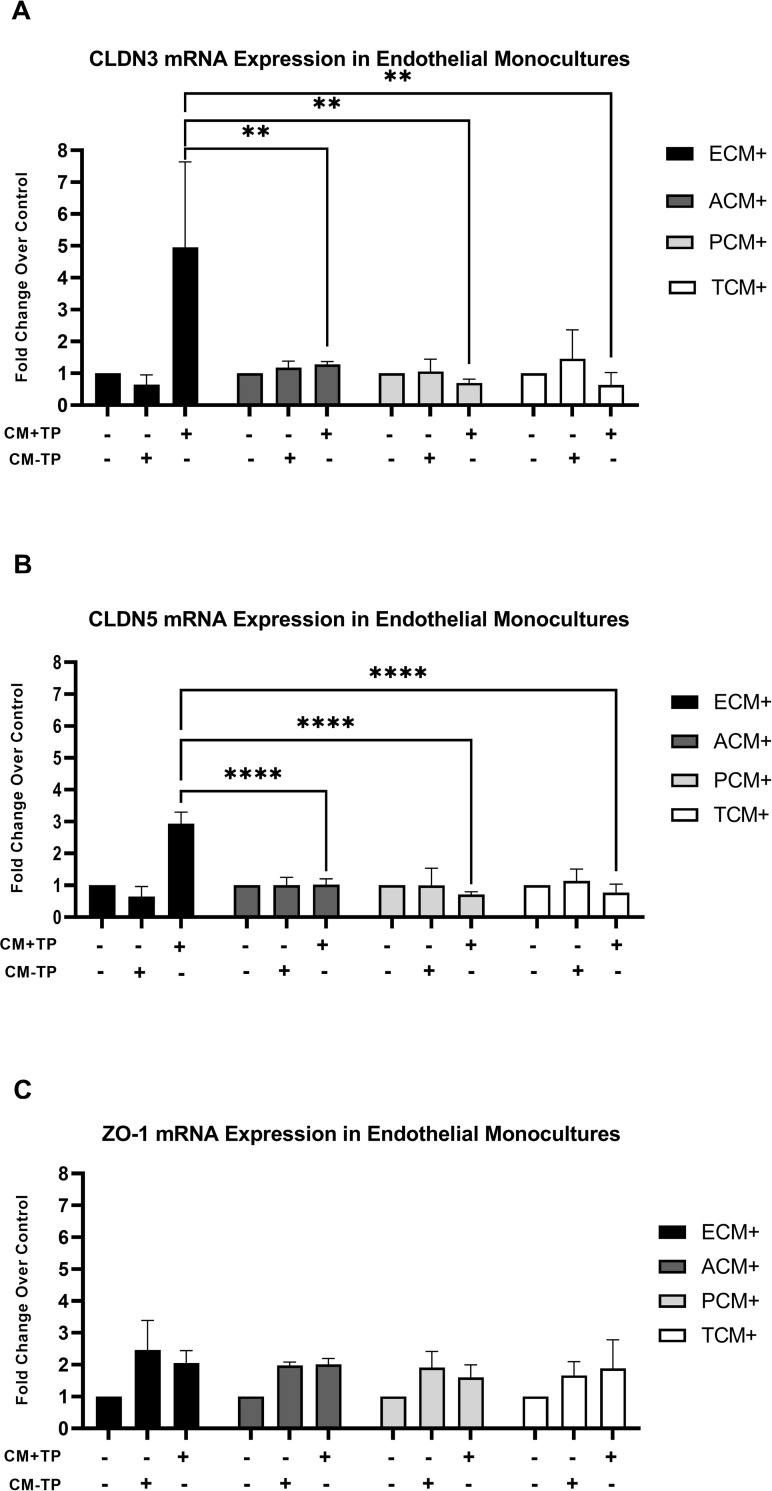
CLDN3, CLDN5, and ZO-1 mRNA expression in endothelial cell monocultures following a 4 hour incubation with cell type specific conditioned media. Endothelial Monoculture Conditioned Media, (ECM+), Astrocyte Monoculture Conditioned Media, (ACM+), Pericyte Monoculture Conditioned Media, (PCM+), and Triculture Conditioned Media, (TCM+), were prepared by either a 1-hour incubation with 5% severely injured patient plasma (CM+TP) or left as is with no plasma addition (CM-TP). All conditioned media was added to endothelial cell monocultures activated with severely injured patient plasma to a working concentration of 5%. Final working concentration of severely injured patient plasma and conditioned media in endothelial monocultures receiving conditioned media was 5% and 10% respectively. Each condition was compared to an endothelial cell monoculture naïve control (CM+TP—and CM-TP -). CM+TP wells were compared against one another to determine the differential effect each cell type’s conditioned media had. (A) CLDN3 mRNA expression: The endothelial monoculture receiving ECM+/CM+TP showed a 4.96-fold change increase vs. control, p<0.01, n = 2; ECM+/CM+TP produced a significant fold change increase in CLDN3 mRNA expression compared to ACM+/ CM+TP, (p<0.01), PCM+/CM+TP, (p<0.01), and TCM+/CM+TP, (p<0.01). Endothelial monocultures receiving ACM+, PCM+, or TCM+ did not show significant fold change vs. control for either CM-TP or CM+TP. Additionally, endothelial monocultures receiving ECM+/CM-TP showed no significant change in transcription. (B) CLDN5 mRNA expression: The Endothelial monoculture ECM+/CM+TP showed a 2.94-fold change increase vs. control, p<0.0001, n = 2; ECM+/CM+TP produced a significant fold change increase in CLDN5 mRNA expression compared to ACM+/CM+TP, (p<0.0001), PCM+/CM+TP, (p<0.0001), and TCM+/CM+TP (p<0.0001). Endothelial monocultures receiving ACM+, PCM+, or TCM+ did not show significant fold change vs. control for either CM+TP or CM-TP. Endothelial monocultures receiving ECM+/CM-TP showed no significant change in transcription. (C) ZO-1 mRNA expression: Endothelial monocultures receiving ACM+, ECM+, PCM+, or TCM+ did not show significant fold change vs. control for either CM+TP or CM-TP. Two experimental replicates of the conditioned media experiment were performed, n = 2. ** Denotes p<0.01. **** Denotes p<0.0001.

A conditioned media exchange study was performed in order to delineate the specific cell types of the neuro vascular unit contributing to transcriptional downregulation of CLDN3 and CLDN5 observed in the triculture model and whether that contribution is due to the release of soluble factors from those cells. (Fig 1A and 1B). The conditioned media exchange studies comprised of the following experimental group. Endothelial monocultures were incubated with conditioned media from a) endothelial monoculture (ECM+), b) astrocytes monoculture (ACM+), c) pericyte monoculture (PCM+) or d) Triculture (TCM+) in the presence or absence severely injured patient plasma. (CM+TP; CM-TP). The transcriptional downregulation of CLDN3 and CLDN5 seen in the triculture model was also observed in endothelial monocultures following transfer of astrocyte and pericyte conditioned media. The endothelial monoculture which received ECM+/CM+TP; showed increased expression of CLDN3 (4.91-fold increase vs. control, p<0.01) and CLDN5 (2.94-fold increase vs. control, p<0.0001) (Fig 2A and 2B). Endothelial monocultures receiving CM-TP: ECM+, ACM+, PCM+, or TCM+ did not show any significant increase in mRNA expression for CLDN3 or CLDN5. (Fig 2A and 2B) Similarly, endothelial monocultures receiving CM+TP: ACM+, PCM+, or TCM+ did not show any significant increase in mRNA expression for CLDN3 and CLDN5. (Fig 2A and 2B) Endothelial monocultures that received either CM+TP or CM-TP: ACM+, PCM+ or TCM+ did not show any significant change in the expression of ZO-1 when compared to controls (Fig 2C).

### Severely injured patient plasma induces differential protein expression of CLDN5

**Fig 3:**
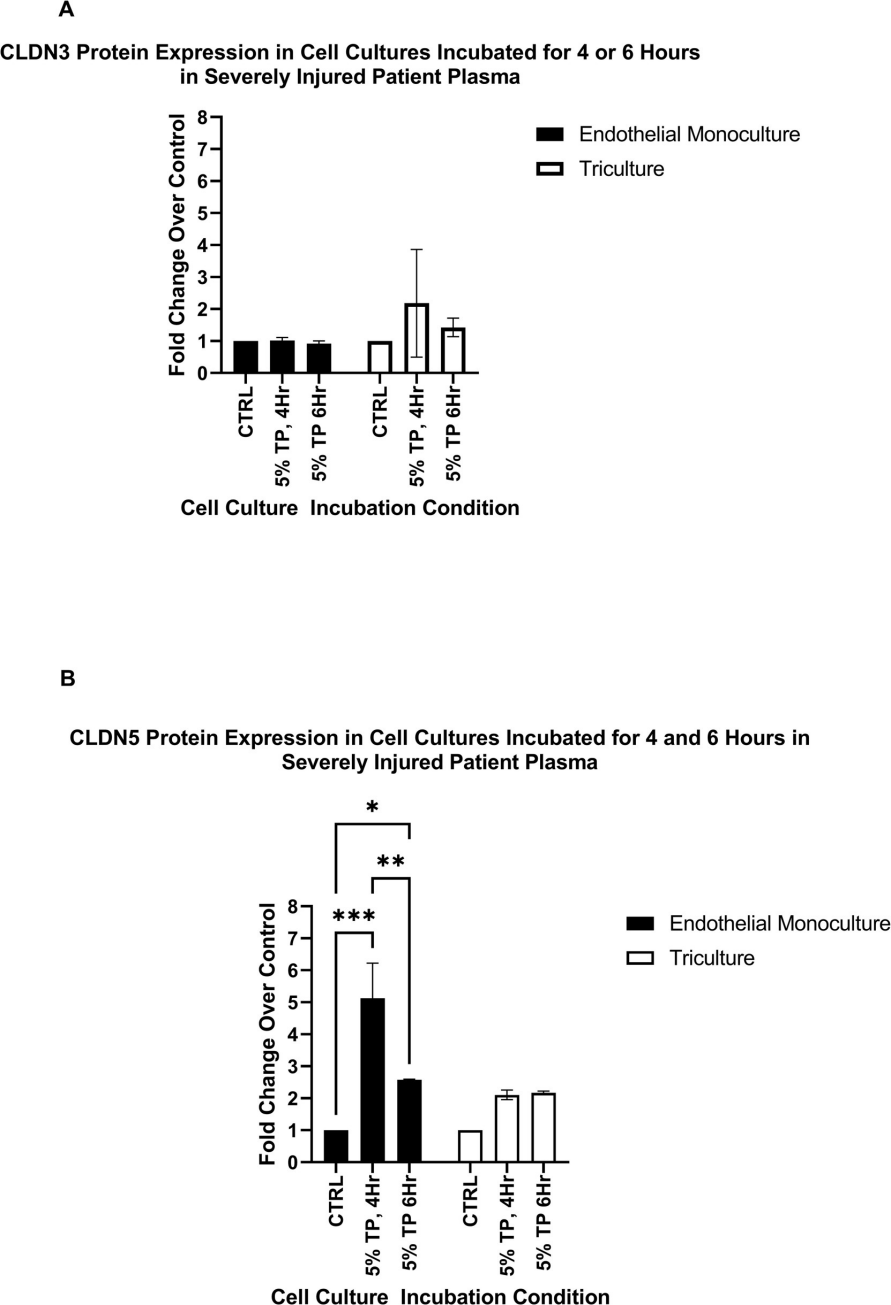
CLDN5, and CLDN3 protein expression following a 4- or 6-hour incubation with 5% plasma from severely injured patients. (A) CLDN3 protein expression: We observed no significant change in fold change of CLDN3 expression was observed in either the endothelial monoculture or triculture, n = 2 (B) CLDN5 protein expression: Endothelial monoculture showed a 5.12- and 2.58-fold change increase vs. control following 4 and 6 hours of incubation respectively with 5% severely injured plasma. p<0.001, p<0.05, n = 2; There is a significant difference between the fold change at 4 hours of incubation compared to 6 hours of incubation following 5% severely injured patient plasma in the endothelial monoculture, p<0.01. The triculture did not show any significant fold change vs. control. One ELISA was performed with the combined whole cell lysate of three experimental replicates, n = 2. * Denotes p<0.05, ** Denotes p<0.01, *** Denotes p<0.001.

In order to determine if the changes in the transcriptional response of the tight junction proteins CLDN3 and CLDN5 in the endothelial monoculture and triculture were also reflected in protein translation, sandwich ELISAs were performed. The pooled plasma used in the translation studies had a mean ISS of 35.6 and BD of -18.36. The protein expression of CLDN3 and CLDN5 were assayed in whole cell lysate. The Endothelial monocultures show a significant increase in CLDN5 protein expression following both a 4 hour incubation (5.12 fold increase vs. control, p<0.001) and 6 hour incubation (2.58 fold change vs. control, p<0.01) with 5% severely injured patient plasma. (Fig 3B) We observed no significant fold changes occurred for CLDN5 protein expression in the Triculture, nor were there any significant fold changes in CLDN3 protein expression for either the endothelial monoculture or triculture (Fig 3A and 3B).

### Addition of astrocyte or pericyte conditioned media suppress endothelial monoculture protein upregulation of CLDN5

**Fig 4:**
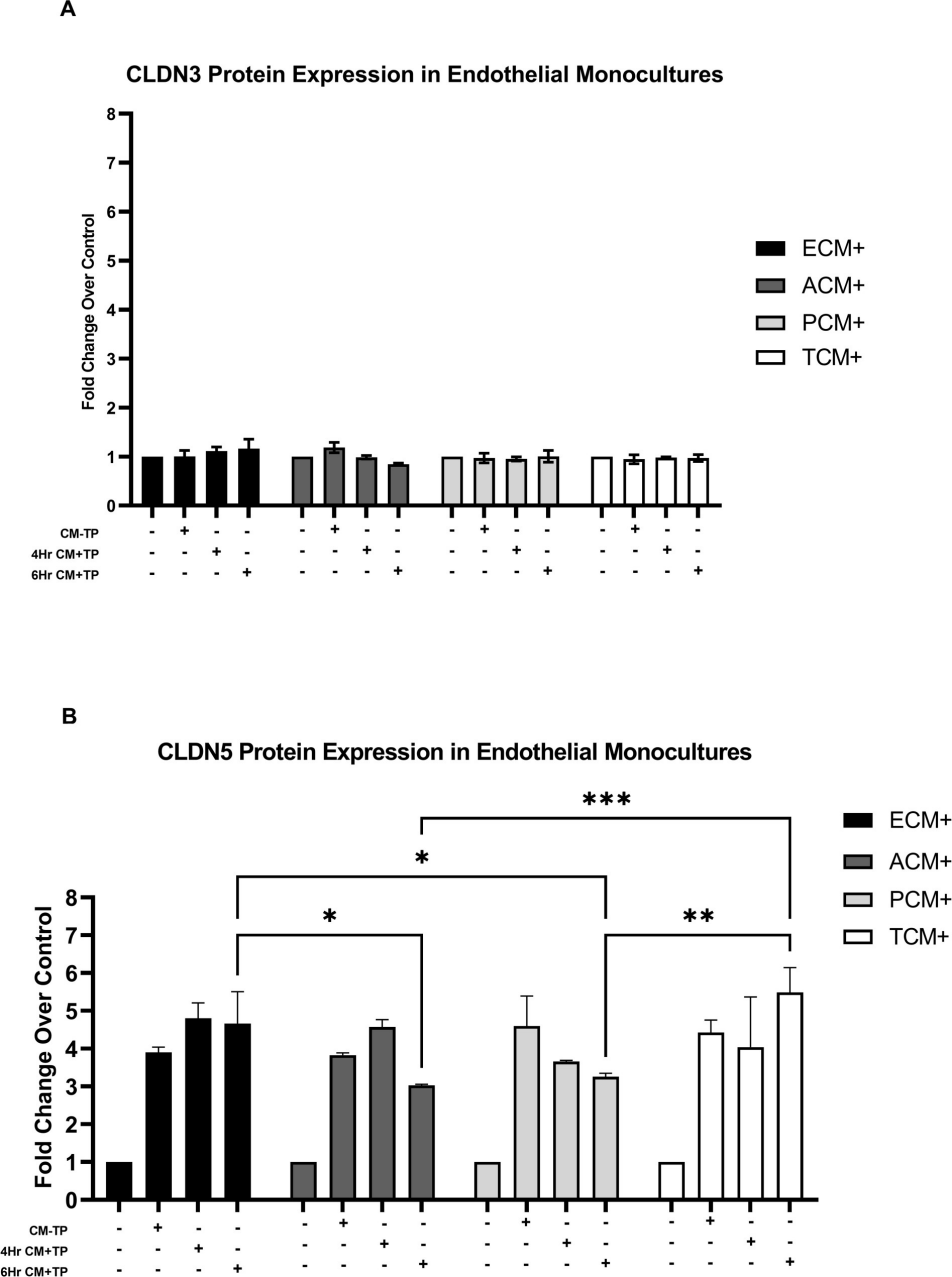
CLDN3 and CLDN5 protein expression in endothelial cell monocultures following a transfer of conditioned. Endothelial Monoculture Conditioned Media, (ECM+), Astrocyte Monoculture Conditioned Media, (ACM+), Pericyte Monoculture Conditioned Media, (PCM+), and Triculture Conditioned Media, (TCM+), were prepared by either a 1 hour incubation with 5% severely injured patient plasma (CM+TP) or left as is with no plasma additions made (CM-TP). Final working concentration of severely injured patient plasma and conditioned media in endothelial monocultures receiving conditioned media was 5% and 10% respectively. Each condition was compared to its Naïve control (CM+TP—and CM-TP -) receiving no conditioned media and no severely injured patient plasma to produce fold changes. CM+TP were compared against one another to determine the differential effect amongst the conditioned media types. Both 4 and 6 hour incubation times were assessed. * Denotes p<0.05, ** Denotes p<0.01, *** Denotes p<0.001 (9) CLDN3 protein expression: Endothelial monocultures did not present with any significant changes in CLDN3 protein expression for all conditions. (10) CLDN5 protein expression: All conditions tested resulted in a significant increase in the fold change of CLDN5 expression compared to the naïve control (not shown). The Endothelial monoculture receiving 6Hr ECM+/CM+TP showed a significant increase in fold change vs. control compared to 6Hr ACM+/CM+TP (p<0.01), and 6Hr PCM+/CM+TP (p<0.05) n = 2; The endothelial monoculture receiving 6Hr TCM+/CM+TP showed a significant increase in fold change compared to 6Hr ACM+/CM+TP (p<0.001), and 6Hr PCM+/CM+TP (p<0.001) n = 2.

The translation of CLDN3 and CLDN5 proteins from the mRNA transcripts from endothelial monocultures following conditioned media exchange was assessed from whole cell lysate. The conditioned media exchange studies had the same experimental setup as the previous media exchange experiment (Fig 2) We observed no significant difference between the condition types or time points for any tested sample for CLDN3 protein expression. (Fig 4A) Endothelial monocultures receiving 6 hour ECM+/CM+TP presented with a significant increase in CLDN5 protein expression compared to endothelial monocultures receiving 6Hr ACM+/CM+TP (4.67 vs 3.03-fold change vs. control respectively, p<0.01) and 6Hr PCM+/CM+TP (4.67 vs. 3.26-fold change vs. control respectively, p< 0.05). (Fig 4B) Endothelial monocultures receiving 6Hr TCM+/CM+TP presented with a significant increase in CLDN5 protein expression compared to endothelial monocultures receiving 6Hr ACM+/CM+TP (5.48 vs 3.03-fold change vs. control respectively, p<0.001) and 6Hr PCM+/CM+TP (5.48 vs. 3.26-fold change vs. control respectively, p<0.001). (Fig 4B) We observed no significant difference between the condition types or time points for any other tested sample (Fig 4B).

## Discussion

Tight junction proteins are central to the maintenance of BBB integrity and increases in BBB permeability have a significant impact on patient outcomes [4,6]. BBB dysfunction has had little inquiry in the context of non-TBI critically injured patients despite their presentation of clinical sequela of brain injury. We demonstrate here that ex vivo plasma from severely injured non-TBI patients causes BBB breakdown accompanied by transcriptional and translational responses of central tight junction proteins of the neurovascular unit required to maintain high resistance across the BBB. Our data shows that the causes of non-TBI injured patient’s presentation of BBB dysfunction center on the expression, or lack thereof, of tight junction proteins key to BBB permeability.

CLDN3, CLDN5, and ZO-1 are central to regulation of BBB permeability [16,17,25–29], and are essential in maintaining barrier integrity [7–9]. Loss of expression of these tight junction proteins compromises barrier integrity by increasing transcellular permeability across the vasculature. Claudin 5 is implicated as a singular driver in multiple disease states including some neuroinflammation states [17] and plays major roles in disease states from carcinomas to schizophrenia and other endothelial barrier dysfunctions [25–27,30]. Following traumatic injuries, an overall downregulation and dysfunction of CLDN5 is observed, coupled directly with BBB permeability dysfunction [4,28,31–33]. There is also evidence that increased exogenous CLDN5 expression may increase the tightness of the junctions [8,33].

This made CLDN5 the primary target of interest for the transcriptional regulation among the three main cell types of the BBB following incubation with severely injured patient plasma. The importance of Claudin 3 as a major tight junction protein and its role in BBB permeability is not yet clearly defined [16,34]. However, because CLDN3 is present in the tight junction of brain endothelial cells and CLDN3’s definitive role in the BBB remains unclear, CLDN3 was chosen as an ideal candidate to focus on in an *in vitro* traumatic injury model. Finally, ZO-1 was analyzed because of its central role in the anchoring of tight junction proteins to the actin filaments. ZO-1’s central role in facilitating foundational structure to tight junctions, coupled with BBB permeability disruptions following its re-localization or degradation from MMPs following injury, made ZO-1 an ideal candidate to include in this study [29,35].

Our studies using an *in vitro* triculture model suggest inhibitory signals from astrocytes and pericytes induce transcriptional downregulation of CLDN3 and CLDN5 and translational downregulation of CLDN5 in brain endothelial cells within four hours of exposure to severely injured patient plasma. (Figs 1A, 1B and 4B) Furthermore, our observations suggest that astrocytes and pericytes in separate monocultures release sufficient soluble factors to elicit inhibition of tight junction expression in endothelial cells. These observations were upheld when endothelial cell monocultures were treated with conditioned media from monocultures of astrocyte or pericytes incubated with 5% severely injured patient plasma. (Figs 2A, 2B, 4A and 4B) The observed inhibition of the tight junction proteins within the triculture following incubation for four hours in severely injured patient plasma is specific to the transcription of CLDN3 and CLDN5 and the translation of CLDN5. We also demonstrate that the use of a healthy plasma negative controls made no significant impact on tight junction expression compared to our naïve control. Due to the insignificance of our healthy plasma’s impact on tight junction expression, the decision was made, with both experimental design and cost in mind, to exclude this condition from future trials. Likewise, ZO-1 showed no differences in transcriptional response (Figs 1C and 2C), leading to its exclusion from translation studies. Finally, CLDN3 expression did not show a robust translational response, as seen with CLDN5, compared to the observed transcriptional changes. (Fig 3A). This does not necessarily decrease the possibility of astrocyte or pericyte derived initial inhibitory signaling leading to decreased endothelial cell tight junction expression; but could support literature that suggests CLDN3 plays a less significant role in restoration and/or maintenance of BBB permeability than that of other tight junction proteins [36].

We observed a robust and significant transcriptional and translational expression of CLDN5 in endothelial monocultures following incubation with severely injured patient plasma which was not observed in tricultures incubated with severely injured plasma. The finding that plasma from severely injured patients downregulates expression of critical tight junction proteins within a triculture model is compelling and is suggestive of a critical role that pericytes and astrocytes play in normal physiological responses to severe traumatic injury via initiation of inhibitory cross talk between the three cell types. This inhibition of CLDN5 expression is further supported by conditioned media experiments. Endothelial monocultures which received conditioned media from astrocytes and pericytes following 6 hours of incubation were observed to have significantly less CLDN5 protein expression compared to endothelial monocultures receiving conditioned media from endothelial monocultures or the triculture. A possible explanation for the inhibition of CLDN5 protein expression in endothelial monocultures receiving astrocyte conditioned media that was not observed in endothelial monocultures receiving triculture conditioned media is unmitigated release of Angiopoietin 2 (ANG2) by activated astrocytes. ANG2, a soluble mediator and a marker of endothelial dysfunction [37], is present in copious amounts in trauma plasma and is associated with worse clinical outcomes through destabilization of endothelial barriers, increasing leakage and edema. ANG2 acts as an antagonist to Angiopoietin 1 (ANG1) for its primary ligand, the receptor tyrosine kinase TIE2 [38]. TIE2 activation leads to dimerization and auto-phosphorylation of its intracellular domain, subsequently activating the PI3K/AKT pathway [39–41]. Inactivation of the PI3K/AKT pathway via ANG2 leads to downregulation of CLDN5 expression through recruitment of a repressor complex to CLDN5’s silencer binding domain [41,42]. While this mechanism is understood in the context of TBI and other pathologies, it is unknown what mechanism causes BBB dysfunction due to non-TBI trauma; but it is suggestive from literature that increased ANG2 expression following injury does contribute in some capacity. This underlying mechanism for the observed CLDN5 transcriptional and translational results will be analyzed in follow-up studies probing the causal link, if any, between ANG2 signaling and non-TBI trauma related BBB dysfunction.

## Conclusion and future direction

Clinical observations by physicians treating non-TBI traumatically injured patients suggest TBI like BBB dysfunction occurs regardless of injury location but correlates directly with level of injury and shock; reinforcing the need to understand the underpinnings BBB dysfunction [43]. Our findings lend a possible explanation for non-brain injury trauma presenting with similar symptoms to TBI by affecting the expression of tight junction central to maintenance of BBB integrity within the neurovascular unit.

Permeability increases following neuroinflammatory stimuli [44,45]. An emerging body of evidence suggests pathology after TBI is driven by alterations in cellular crosstalk between endothelial cells astrocytes and pericytes [46] via the release of multiple biomolecules of interest including angiopoietin 2, endothelin-1, tumor necrosis factor- alpha, and matrix metalloprotease-9 [33,46–50]. The impact of these molecular mediators and proteases on tight junction expression following trauma, and the subsequent role this modulation of tight junction expression has on BBB permeability, is not yet fully understood.

Our data is consistent with a physiological process designed to prevent infection and maximize repair of cellular structures within the interneuronal space [8,15,50–53]. Delaying BBB permeability restoration through the inhibition of tight junction proteins activate physiological responses to neuroinflammatory factors such as TNF-alpha, IL1-B, IL-6 and other cytokines released during injury and allows for the localization and infiltration of leukocytes across the barrier through ICAM and VCAM dependent processes [51,52]. This infiltration of leukocytes coupled with increased flow of other soluble factors between the vasculature and interneuronal space may provide better neuronal repair following minor brain injuries [8,15,51]. However, when the injury is more severe, this delay in endothelial barrier restoration prevents neurologic recovery and may lead to secondary brain injury. Our results, while observed in a physiological presentation closer to that of the BBB than other *in vitro* models, requires future *in vivo* work to strengthen these findings. This is due to inherent limitations of *in vitro* studies such as lack of shear stress flow, absence of glial cells the complexities of the microenvironment the cells of the neurovascular unit encounter [54,55]. We do believe, however, that isolating the cells *in vitro* gives a better controlled environment to probe the exact cellular response to specific conditions introduced via the severely injured patient plasma that cannot be controlled for *in vivo* [55].

The underlying molecular mechanisms leading to increased blood brain permeability following traumatic injury are poorly enumerated and a comprehensive understanding of this process would provide novel approaches in designing future interventions to prevent the adverse effects of secondary brain injury. The findings in this study will guide our future work to include transcriptional and translational analysis of other tight junction proteins central to BBB permeability and identify soluble factors in plasma that cause perturbations in tight junction transcription and translation. We expect that enhanced understanding of this astrocyte, pericyte, and endothelial cellular crosstalk will help identify new therapies for BBB pathologies following trauma and will provide useful insight in designing specific interventional strategies in patients with severe BBB dysfunction while creating a path forward for implementation of these therapies in personalized healthcare for traumatically injured patients.

## Supporting information

S1 Data Underlying data and statistics used to generate Fig 1. (XLSX)

S2 Data Underlying data and statistics used to generate Fig 2. (XLSX)

S3 Data Underlying data and statistics used to generate Fig 3. (XLSX)

S4 Data Underlying data and statistics used to generate Fig 4. (XLSX)
